# Genomic Analysis of Visceral Fat Accumulation in Holstein Cows

**DOI:** 10.3389/fgene.2021.803216

**Published:** 2022-01-04

**Authors:** Larissa C. Novo, Ligia Cavani, Pablo Pinedo, Pedro Melendez, Francisco Peñagaricano

**Affiliations:** ^1^ Department of Animal and Dairy Sciences, University of Wisconsin, Madison, WI, United States; ^2^ Department of Animal Sciences, Colorado State University, Fort Collins, CO, United States; ^3^ School of Veterinary Medicine, Texas Tech University, Amarillo, TX, United States

**Keywords:** abdominal fat, genomic scan, fat deposition, metabolic disorders, displaced abomasum

## Abstract

Visceral fat is related to important metabolic processes, including insulin sensitivity and lipid mobilization. The goal of this study was to identify individual genes, pathways, and molecular processes implicated in visceral fat deposition in dairy cows. Data from 172 genotyped Holstein cows classified at slaughterhouse as having low (*n* = 77; omental fold < 5 mm in thickness and minimum fat deposition in omentum) or high (*n* = 95; omental fold ≥ 20 mm in thickness and marked fat deposition in omentum) omental fat were analyzed. The identification of regions with significant additive and non-additive genetic effects was performed using a two-step mixed model-based approach. Genomic scans were followed by gene-set analyses in order to reveal the genetic mechanisms controlling abdominal obesity. The association mapping revealed four regions located on BTA19, BTA20 and BTA24 with significant additive effects. These regions harbor genes, such as *SMAD7*, *ANKRD55*, and the HOXB family, that are implicated in lipolysis and insulin tolerance. Three regions located on BTA1, BTA13, and BTA24 showed marked non-additive effects. These regions harbor genes *MRAP, MIS18A*, *PRNP* and *TSHZ1,* that are directly implicated in adipocyte differentiation, lipid metabolism, and insulin sensitivity. The gene-set analysis revealed functional terms related to cell arrangement, cell metabolism, cell proliferation, cell signaling, immune response, lipid metabolism, and membrane permeability, among other functions. We further evaluated the genetic link between visceral fat and two metabolic disorders, ketosis, and displaced abomasum. For this, we analyzed 28k records of incidence of metabolic disorders from 14k cows across lactations using a single-step genomic BLUP approach. Notably, the region on BTA20 significantly associated with visceral fat deposition was also associated with the incidence of displaced abomasum. Overall, our findings suggest that visceral fat deposition in dairy cows is controlled by both additive and non-additive effects. We detected at least one region with marked pleiotropic effects affecting both visceral fat accumulation and displaced abomasum.

## Introduction

Fat deposition occurs essentially in three regions, namely intramuscular, subcutaneous, and around visceral organs. These fat depots defer in structural organization, cellular size, biological function, and immunological and metabolic characteristics. Historically, visceral fat was considered to protect and insulate the internal organs; however, its critical role as a form of energy storage and endocrinological signaling has been recently recognized (Booth et al., 2016). Indeed, visceral adipose tissue carries out relevant functions, including lipogenesis to store surplus energy as triglycerides during periods of overnutrition, lipolysis to release energy as free fatty acids during periods of undernutrition, and secretion of a broad spectrum of active molecules, such as proinflammatory cytokines (Item and Konrad, 2012). Moreover, visceral adiposity has been linked to several metabolic disorders, including impaired glucose and lipid metabolism, and insulin resistance (Konrad and Wueest, 2014).

Dairy cows typically experience a state of negative energy balance around parturition and early lactation, when the energy demand for maintenance and milk production exceeds that of dietary energy intake (Pascottini et al., 2020). This negative energy balance leads to fat mobilization, and consequently, an increase in plasma concentrations of non-esterified fatty acids (NEFA), which are used as a fuel source by peripheral tissues and the mammary gland for milk fat synthesis. Visceral adipocytes in dairy cows are more metabolically active and sensitive to lipolysis than subcutaneous adipocytes, and hence, visceral adipose tissue is a key player in metabolic health during the transition period (Ji et al., 2014b). Note that extreme lipid mobilization is associated with different metabolic disorders, including displacement of the abomasum, ketosis, and fatty liver (Hostens et al., 2012; Contreras et al., 2015). Displacement of the abomasum, a condition in which the abomasum becomes enlarged with fluid and gas with subsequent migration to the left or right within the abdominal cavity, results in reduced rumination, decreased milk production, increased veterinary costs, and premature culling.

There is growing evidence that body fat distribution is influenced by genetic factors. For instance, in humans, waist-hip ratio, a measure of body fat distribution independent of overall adiposity, is a heritable trait controlled by multiple significant loci (Heid et al., 2010). Recently, Melendez et al. (2018) provided the first genetic report for visceral fat accumulation in dairy cows. These authors found that visceral fat deposition is a heritable trait, and hence, its prevalence in dairy cattle could be reduced by genetic selection. They also found evidence that variation in genes involved in preadipocyte signaling and adipocyte development could explain part of the differences observed in visceral fat accumulation.

The first objective of this study was to reanalyze the data from Melendez et al. (2018) using alternative methods for gene mapping and the application of SNP-based gene-set enrichment tools. Given that visceral fat accumulation is associated with metabolic health, the second objective of this study was to identify genomic regions with pleotropic effects on visceral adiposity and incidence of ketosis or displaced abomasum.

## Materials and Methods

### Visceral Fat Accumulation

Data were collected at a slaughterhouse located in Green Bay, WI, United States (Melendez et al., 2018). Briefly, adult Holstein cows were evaluated from the processing line. After the carcass was eviscerated, the amount of omental fat at the level of the insertion of the lesser omentum over the pylorus area was assessed. Low visceral fat accumulation was defined as an omental fold <  5 mm in thickness and minimum fat deposition observed throughout the entire omentum. High visceral fat accumulation was defined as an omental fold ≥  20 mm in thickness and marked fat deposition observed throughout the entire omentum. Body condition score was measured using a 5-point scale, and only cows with body condition score between 2.75 and 3.25 were considered. As mentioned by Melendez et al. (2018), the goal of this sampling protocol was to obtain two groups of Holstein cows with extreme differences in visceral fat accumulation but with very similar subcutaneous fat deposition. A total of 172 cows were finally selected for this study, 77 with low and 95 with high visceral fat accumulation.

These 172 cows were genotyped using the Illumina BovineHD Beadchip with over 777k single nucleotide polymorphism (SNP) probes. After removing monomorphic markers and those located in the X chromosome, a total of 584,557 SNPs remained for the genomic analyses.

### Genomic Scans

The importance of additive and non-additive effects on visceral fat accumulation was evaluated using a two-step mixed-model-based approach (Aulchenko et al., 2007).

In the first step, the following model was fitted:

y = Xb+Zu + e

where y is the vector of visceral fat accumulation scores (low = 0 or high = 1), b is the vector of fixed effects, u is the vector of random animal effects, and e is the vector of random residual effects. The incidence matrices X and Z relate visceral fat accumulation scores to fixed and animal effects, respectively. The random effects were assumed to follow a multivariate normal distribution with $u∼N\left(0,G\sigma_{u}^{2}\right)$ and $e∼N\left(0,I\sigma_{e}^{2}\right)$ , where $\sigma_{u}^{2}$ and $\sigma_{e}^{2}$ are the animal additive genetic and residual variances respectively, G is the genomic relationship matrix, and I an identity matrix. The variance-covariance matrix for this first model was estimated as $V_{0}=\;\mathrm{ZG}Z^{\prime }\sigma_{u}^{2}+I\sigma_{e}^{2}$ .

In the second step, the following model was fitted for every SNP:

$$y=\;\mathrm{X\beta}\;+\;X_{SNP}\beta_{SNP}\;+\;\epsilon_{\;}^{\;}$$

where $X_{SNP}$ is the design matrix for the marker under consideration and $\;\beta_{SNP}$ is the regression coefficient, also known as SNP effect. Every SNP genotype was coded using single numeric variables as (0, 1, 2), (0, 1, 1), (0, 0, 1) and (0, 1, 0) for testing additive, dominance, recessive and overdominance effects, respectively. This model assumes that $\epsilon ∼N\left(0,V_{0}\sigma_{e}^{2}\right)$ . The significance of each SNP effect was evaluated using the following test statistic:

$$z=\frac{X\prime_{SNP}V_{0}^{-1}\left(y-X\hat{\text{β}}\right)}{\sqrt{X\prime_{SNP}V_{0}^{-1}X_{SNP}}}$$

Which approximates the Wald test, and hence, is asymptotically standard normal. These analyses were performed using the R package MixABEL (Aulchenko et al., 2007). The possible inflation of the test statistics was evaluated using quantile-quantile (Q-Q) plots.

### Overrepresentation Analysis

The overrepresentation analysis, also known as gene-set analysis, was performed in three steps as described by Han and Peñagaricano (2016). The first step was the assignment of SNP markers to annotated genes. The latest bovine reference annotation (ARS-UCD1.2) was used to retrieve the exact location of each annotated bovine gene in the genome. SNP markers were assigned to annotated genes if they were located within the genomic sequence of the gene or at most 10 kb upstream or downstream the gene. Significant genes were defined as those genes containing at least one SNP with a significant additive effect (*p*-value ≤ 0.01). The second step was the assignment of annotated genes to gene-sets. Six different gene-set databases were explored, including GO, KEGG, MeSH, InterPro, MSigDB, and Reactome. Finally, in the third step, the enrichment or overrepresentation of significant genes in each gene-set was tested using a Fisher’s exact test. All these analyses were performed using the *R* package EnrichKit (https://github.com/liulihe954/EnrichKit), developed by our group.

### Genomic Analysis of Metabolic Disorders

Data consisted of producer-recorded lactation incidence records of two important metabolic disorders, namely displaced abomasum and ketosis, collected in one large commercial dairy herd in the state of Florida, USA. A total of 27,408 records of displaced abomasum from 13,401 cows and 26,752 records of ketosis from 13,133 cows were collected between January 2010 and December 2015. Metabolic disorders were recorded as binary, i.e., Y = 1 if the cow had clinical symptoms, and Y = 0 otherwise. A total of 487 and 2393 cows had at least one case of displaced abomasum or ketosis, respectively. The incidence of displaced abomasum was 1.8% (491 cases) while the incidence of ketosis was 10.4% (2772 cases). Genotype data for 60,671 single nucleotide polymorphism (SNP) markers were available for 5.9k cows with health records and 1.4k sires in the pedigree. Markers that mapped to the sex chromosomes, or were monomorphic, or had minor allele frequency less than 1% were removed from the SNP dataset. After data editing, a total of 54,043 SNPs were retained for subsequent analyses.

The incidence of metabolic disorders was analyzed using a threshold model (Gianola, 1982). This model, also known as probit model, describes the observable response variable (0 or 1) using an underlying linear model, z=η+ε **,** where η is a vector of linear predictors and ε is a vector of independent and identically distributed standard normal random variables. Here, the liner predictor η had the following form:

$$\eta \;=\;\mathrm{X\beta}\;+\;Z_{1}\mathrm{hys}+\;Z_{2}u+\;\mathrm{Wpe}$$

where β is a vector of fixed effects in the model, hys is a vector of random herd-year-season effects, u is a vector of random additive genetic effects, and pe is a vector of random permanent environmental effects. The vector β includes the intercept and the lactation number as a class variable with 5 levels (1, 2, 3, 4, and 5+). The matrices X , $Z_{1}$ , $Z_{2}$ , and W are the incidence matrices relating health records to fixed, herd-year-season, animal, and permanent environmental effects, respectively. The random effects were assumed to follow a multivariate normal distribution with $\mathrm{hys}∼N\left(0,I\sigma_{hys}^{2}\right)$ , $u∼N\left(0,H\sigma_{u}^{2}\right)$ , and $\mathrm{pe}∼N\left(0,I\sigma_{pe}^{2}\right)$ , where $\sigma_{hys}^{2}$ , $\sigma_{u}^{2}$ and $\sigma_{pe}^{2}$ are the hear-year-season, animal additive genetic, and permanent environmental variances respectively, H is a relationship matrix, and I an identity matrix. The matrix H combines pedigree and genotypic information (Aguilar et al., 2010).

Candidate genomic regions associated with metabolic disorders were identified based on the amount of genetic variance explained by 2.0 Mb window of adjacent SNPs. The SNP effects were estimated as $\hat{s}=\mathrm{DM}\prime {\left[\mathrm{MDM}\prime \right]}^{-1}{\hat{a}}_{g}$ , where $\hat{s}$ is the vector of SNP marker effects, D is a diagonal matrix of weights of SNPs, **M** is a matrix relating genotypes of each SNP marker to observations, and ${\hat{a}}_{g}$ is the vector of genomic estimated breeding values for genotyped animals (Wang et al., 2012). The percentage of genetic variance explained by a 2.0 Mb region was calculated as,

$$\frac{Var\left(u_{i}\right)}{\sigma_{u}^{2}}\times 100=\frac{Var\left(\sum_{j=1}^{N}M_{j}s_{j}\right)}{\sigma_{u}^{2}}\times 100$$

where $u_{i}$ is the genetic value of the $i^{th}$ genomic region under consideration, N  is the total number of adjacent SNPs within the 2.0 Mb region, $M_{j}$ is the vector of SNP content of the *j* ^*th*^SNP for all individuals, and $s_{j}$ is the marker effect of the $j^{th}$ SNP within the $i^{th}$ region. These analyses were performed using the program POSTGSF90 (Aguilar et al., 2014).

## Results

### Genomic Scans for Visceral Fat Accumulation

**FIGURE 1:**
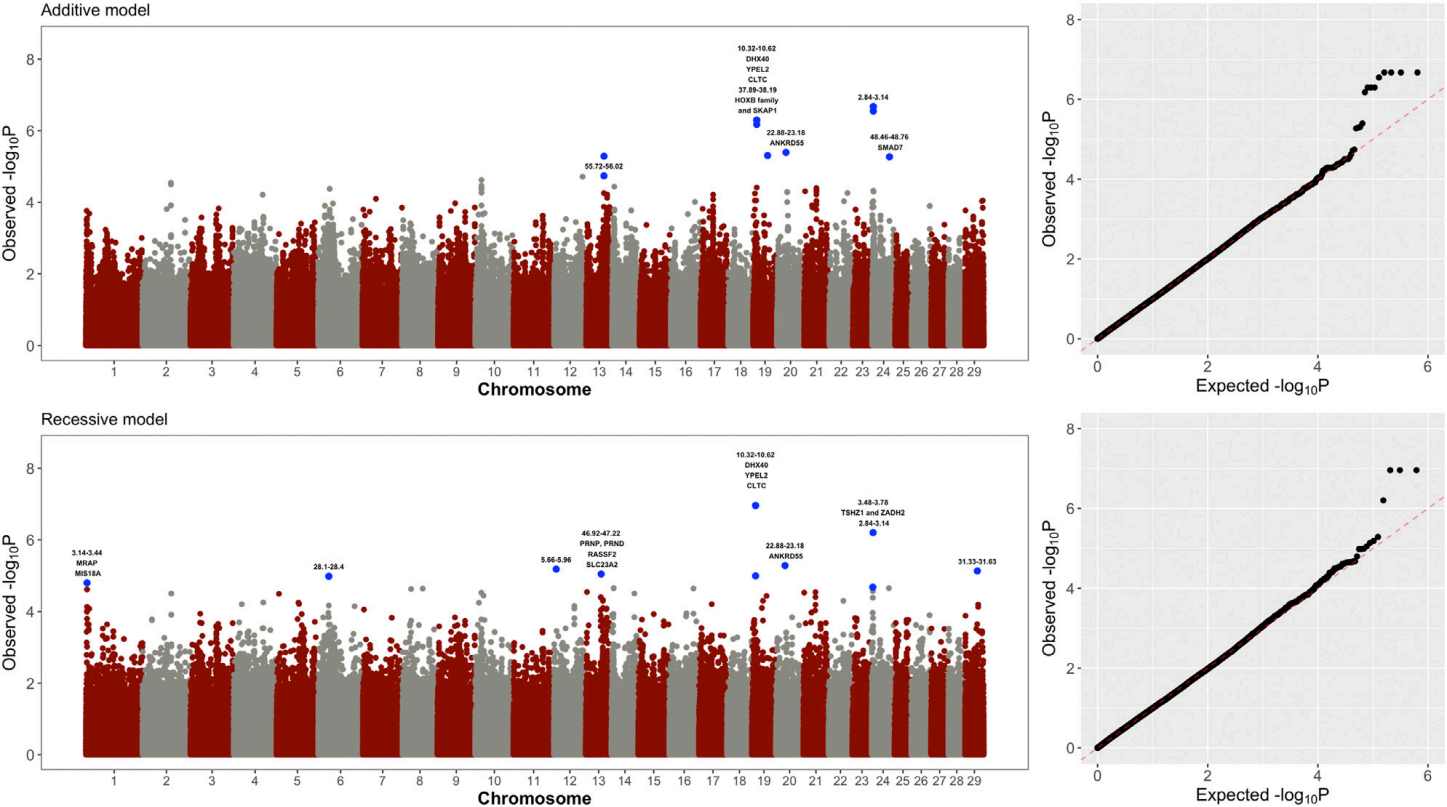
Manhattan plots and quantile-quantile plots showing the significance of additive and recessive effects on visceral fat accumulation across the entire bovine genome. Genes directly implicated in adipocyte differentiation, lipid metabolism, and insulin sensitivity are highlighted in the Manhattan plots.

Figure 1 shows the results of the whole-genome single marker scans for testing both additive and non-additive (recessive) genetic effects on visceral fat accumulation in Holstein cows. Genomic regions on chromosomes BTA19, BTA20 and BTA24 showed the most significant additive effects (Table 1). The two significant regions in BTA19 (10.32–10.62 Mb and 37.89–38.19 Mb) harbor candidate genes *DHX40*, *YPEL2*, *CLTC*, *SKAP1,* and HOXB1-6 family. These genes are implicated in different functions, including cell proliferation, cell differentiation and immunity. Moreover, the significant region detected in BTA20 (22.88–23.18 Mb) harbors the gene *ANKRD55*, which is related to adipocyte proliferation and lipolysis. Finally, the significant region in BTA24 (48.46–48.76 Mb) harbors the gene *SMAD7*, which is associated with glucose uptake and obesity.

**TABLE 1:** Genetic markers and candidate genes associated with visceral fat accumulation in Holstein cows

Chromosome	Position	*p*-value	Candidate gene	Process
Markers with additive effects				
BTA19	10.32–10.62	5.0e-07	*YPEL2*	Cell cycle
BTA19	37.89–38.19	4.9e-06	*CLTC*	Cell proliferation
		4.9e-06	*HOXB1-6*	Development, morphogenesis
		4.9e-06	*SKAP1*	Adaptive immune response
BTA20	22.88–23.18	4.0e-06	*ANKRD55*	Pre-adipocyte proliferation, differentiation
BTA24	48.46–48.76	5.4e-06	*SMAD7*	Regulation of TGF-β signaling
Markers with non-additive effects				
BTA1	3.14–3.39	2.4e-05	*MRAP*	Lipolysis, energy balance
		2.4e-05	*MIS18A*	Cell cycle, cell proliferation
BTA13	46.92–47.22	8.9e-06	*PRNP*	Adipocyte differentiation
		8.9e-06	*SLC23A2*	Vitamin C transport
BTA24	3.48–3.78	6.3e-07	*TSHZ1*	Pancreatic β-cell maturation
		6.3e-07	*ZADH2*	Adipocyte differentiation

Of particular interest, three genomic regions located in BTA1, BTA13 and BTA24 showed purely non-additive (recessive) effects (Figure 1 and Table 1). The region in BTA1 (3.14–3.39 Mb) harbors the candidate genes *MRAP* and *MIS18A* that are implicated in insulin sensitivity and obesity. The region in BTA13 (46.92–47.22 Mb) harbors the gene *PRNP* which is directly involved in visceral fat adipose tissue deposition. The significant region in BTA24 (3.48–3.78 Mb) harbors the gene *TSHZ1* and *ZADH2* that is associated with adipocyte differentiation and lipid metabolism.

### Overrepresentation Analysis

**FIGURE 2:**
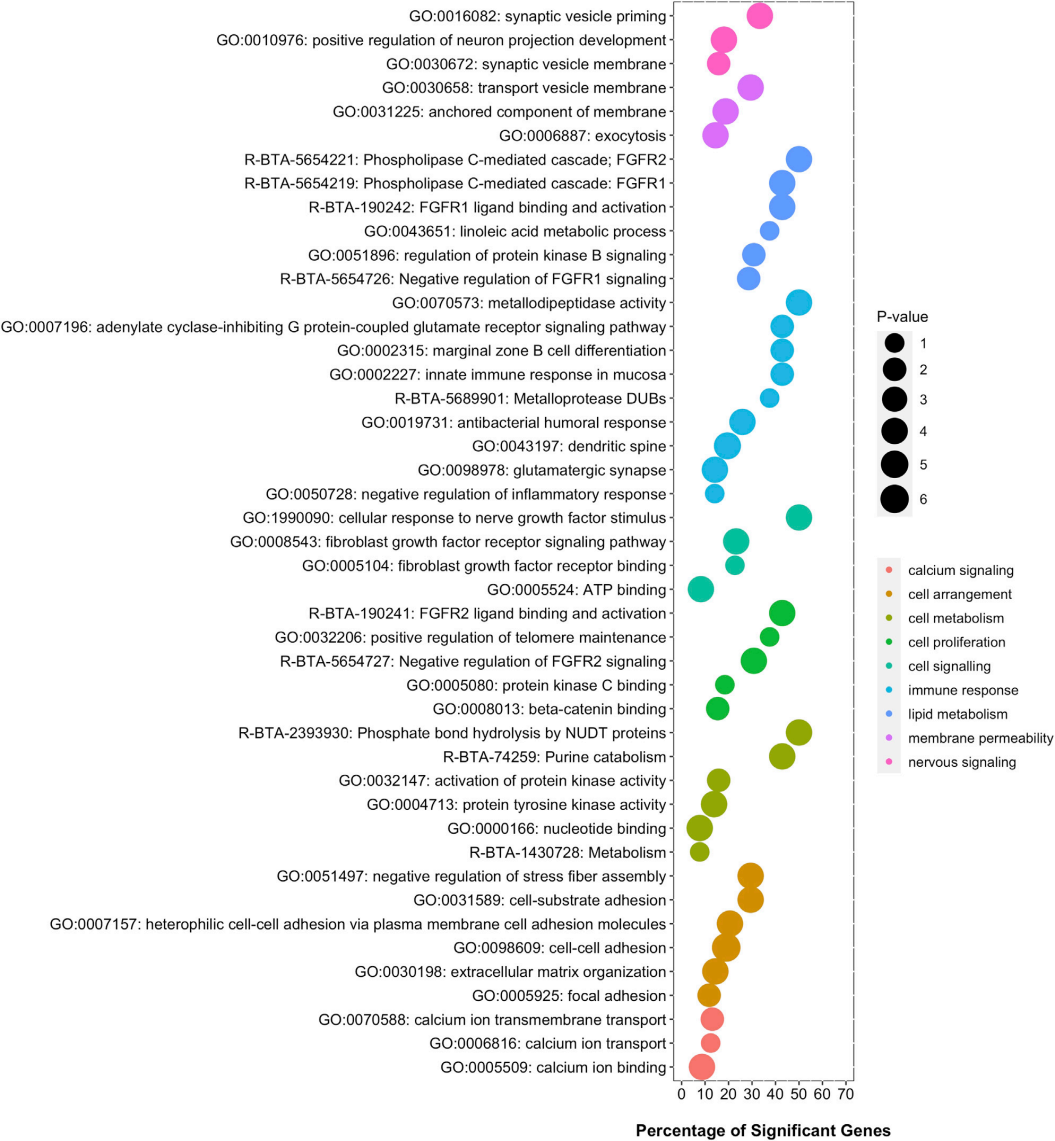
Functional terms significantly enriched with genes associated with visceral fat accumulation. Six gene-set databases were analyzed: GO, KEGG, MeSH, InterPro, MSigDB, and Reactome. The *y*-axis displays the name while the *x*-axis displays the percentage of significant genes in each functional enriched term.

The overrepresentation analysis, namely the search for gene-sets or gene pathways that show an overrepresentation of significant genes, was performed using a Fisher’s exact test, a test of proportions based on the cumulative hypergeometric distribution. Figure 2 shows a set of terms that were significantly enriched with genes associated with visceral fat accumulation. These functional terms are related to calcium signaling, cell arrangement, cell metabolism, cell proliferation, cell signaling, immune response, lipid metabolism, membrane permeability, and nervous signaling, among other functions.

### Genomic Analysis of Metabolic Disorders

**FIGURE 3:**
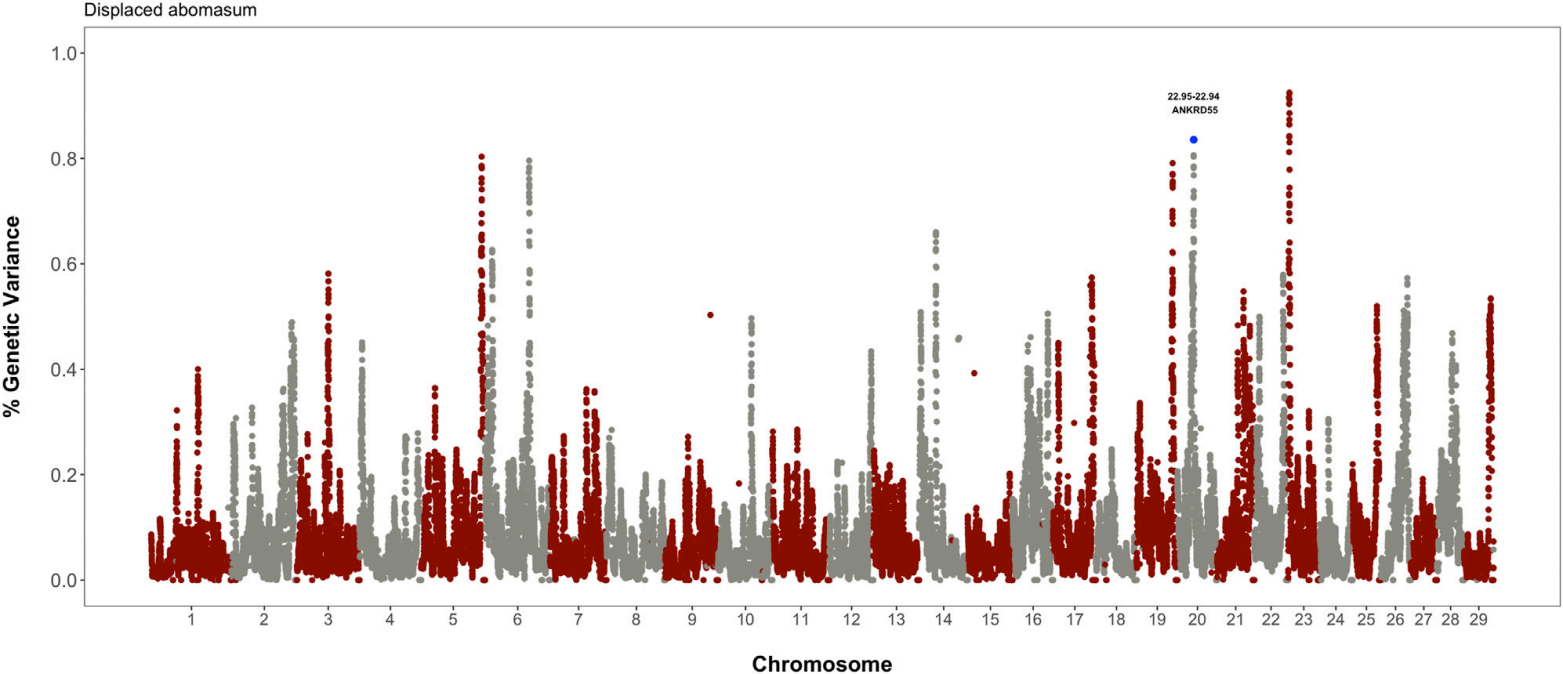
Whole-genome scan for incidence of displaced abomasum: percentage of additive genetic variance explained by 2.0 Mb SNP-windows across the entire bovine genome. Candidate gene *ANKRD55* is implicated in both visceral fat accumulation and displaced abomasum.

The identification of genomic regions affecting displaced abomasum or ketosis was performed using the single-step genomic BLUP. This method combines all the available phenotypic, genotypic, and pedigree information, and fits all the SNP simultaneously. Candidate regions were identified based on the amount of genetic variance explained by 2.0 Mb SNP-windows. Figure 3 shows the gene mapping results for displaced abomasum. Notably, the prominent peak in BTA20, which harbors gene *ANKRD55*, was also significantly associated with visceral fat accumulation, suggesting a pleiotropic action. On the other hand, there were not common regions between ketosis and visceral fat accumulation (data not shown).

## Discussion

Visceral fat is a highly active tissue involved in complex metabolic processes, such as energy supply, inflammation, and insulin sensitivity. Dairy cows with excessive visceral fat are more susceptible to metabolic disorders (Drackley et al., 2014; Ji et al., 2014a; Singh et al., 2014). Although its importance on health and production traits, very few studies have investigated the genetic basis of visceral fat accumulation in dairy cattle. As such, this study was specially conducted to identify individual genes, functional gene-sets and biological pathways associated with visceral fat accretion in Holstein cows. Two groups of cows with extreme differences in fat deposition in the omentum, one of the most important visceral fat depots, but with very similar subcutaneous fat deposition, were evaluated. Note that omental fat is strongly correlated with total visceral fat. Furthermore, given the relationship between visceral fat and metabolic disorders, we also investigated the genetic link between visceral fat accumulation and the incidence of displaced abomasum and ketosis in early lactation.

Several pathways revealed in our study coincide with cell rearrangement of adipose tissue identified in other species in obesity studies. In fact, our gene-set analysis detected biological pathways directly involved in the visceral fat expansion, such as cell arrangement, cell proliferation and cytoskeleton regulation. Additionally, some of the most significant genes detected in our whole-genome scans are directly implicated in lipid accumulation and tissue rearrangement. For instance, gene *PRNP* encodes the cellular prior protein known to regulate visceral fat volume, body fat weight, adipocyte cell size, and body weight gain in mice (Jeong et al., 2019). Similarly, the significant gene *ZADH2*, also known as *PTGR-3,* negatively modulates adipocyte differentiation through regulation of PPARγ, a major regulator of adipogenesis (Yu et al., 2013). Regarding cell rearrangement, the significant gene *YPEL2* is known to be involved in cell division (Hosono et al., 2004) and the HOXB family to encode for transcription factors involved in the anatomical structure of omental fat (Ahn et al., 2019). Genes implicated in the cascade signaling due to the active release of NEFA on liver were also detected. Notably, gene *SMAD7*, one of the most significant genes revealed in the association mapping, is associated with higher levels of circulating NEFA, lower expression of lipolytic genes, and more proinflammatory proteins in obese mice (Seong et al., 2018). While the action of *SMAD7* occurs by downregulating TGF-β pathway, the significant gene *CLTC* stimulates this pathway while downregulate NADPH oxidase to protect against the negative effects of highly active NEFA oxidation (Han, 2016; Caballero-Díaz et al., 2020). Additionally, our overrepresentation analysis detected pathways related to fibroblast growth factor receptors (FGFR), calcium signaling, protein kinases and glutamate signaling. The FGFR1 signaling pathway is related to lipid droplet dynamics, phospholipid homeostasis, protection against oxidative stress and to hypertrophy in obese individuals (Ye et al., 2016). The FGFR2 signaling pathway, inhibited by the significant gene *SMAD7*, indirectly promotes lipid biosynthesis by reducing cAMP pool and protein kinase A (PKA) activity (Ornitz and Itoh, 2015; Huang et al., 2018). Interestingly, both cAMP and PKA are affected by the gene *MRAP*, a significant gene detected in the non-additive scan. Of particular interest, this gene is associated with mitochondrial fatty acid oxidation and is essential for the lipolytic response to adrenocorticoid hormone, and consequently, insulin sensitivity (Zhang et al., 2018).

Research has shown that inflammation is directly impacted by higher availability of glucose and NEFA (Patel and Abate, 2013). Interestingly, our study revealed many genes and gene-sets associated with inflammation. For instance, the gene *NOX4*, the main source of reactive oxygen species (ROS), is highly expressed in adipocytes and is controlled by gene *CLTC*, which was detected as significantly associated with fat accumulation in our whole-genome scan (Den Hartigh et al., 2017). Our enrichment analysis detected calcium transport terms that are known to be affected by ROS in the form of impaired calcium homeostasis that can lead to cell death (Dejos et al., 2020). Other adipose-related inflammation responses were also detected is this work. For instance, the significant gene *SLC23A2* codes for SVCT1, a transporter of vitamin C, a well-known antioxidant that is able to inhibit adipocyte differentiation and lipid accumulation (Rahman et al., 2014). Significant functional terms such as protein kinase C, dendritic spine, and metalloproteinases may indicate the response to local inflammation via proliferation, activation and communication of T-cells, respectively (Black and Black, 2012; Khokha et al., 2013; Sundara Rajan and Longhi, 2016). Interestingly, the gene *SKAP1* identified in the genomic scan is an immune cell adaptor responsible for regulating multiple functions of T-cells (Raab et al., 2019). Enriched terms such as metalloproteases and bacterial humoral defense can also be related to systemic inflammation as the threshold between the beneficial acute inflammation and damaging effect of chronic inflammation is controlled by metalloproteinases via macrophage activity (Khokha et al., 2013).

There is growing evidence that excessive visceral fat may lead to metabolic disorders. In fact, the significant genes and pathways identified in this study suggest a differential inflammatory response among cows with different levels of visceral fat. It is known that cows with displaced abomasum have preferable mobilization of visceral over subcutaneous fat and present higher macrophage infiltration in the omental adipose tissue compared to healthy individuals (Hostens et al., 2012; Contreras et al., 2015). Interestingly, our work revealed one region in chromosome 20 that has significant effects on both visceral fat accumulation and susceptibility to displaced abomasum. Notably, this region harbors the gene *ANKRD55*, which encodes a scaffold protein related to proliferation of pre-adipocytes, insulin sensitivity, and even more important, higher visceral fat accumulation in human subjects (Harder et al., 2013; Ji et al., 2019; Chen et al., 2020). Gene *ANKRD55* is highly active in immune diseases, which corresponds to the state of the clinically diagnosed displaced abomasum cows used in this study, as studies have shown that cows with displaced abomasum are under active lipolysis, negative energy balance, and under higher infiltration of macrophages in adipose tissues (Hostens et al., 2012; Contreras et al., 2015). The whole-body insulin resistance possibly promoted by *ANKRD55* would endorse the insulin resistance stimulated by cytokines release from visceral fat and the impaired glucose-stimulated insulin secretion in pancreatic β cells mediated by the significant gene *TSHZ1* (Raum et al., 2015). Changes in insulin concentration and blood calcium levels, two mechanisms identified in our gene-set analysis, are some of the causes for displaced abomasum in cows (Van Winden et al., 2003). This common peak in BTA20 for visceral fat accumulation and displaced abomasum suggests a genetic link between visceral fat levels and the incidence of metabolic diseases that deserves further investigation.

## Conclusion

In this study, we performed an integrative genomic analysis to better understand the genetic basis of visceral fat accumulation in Holstein cows. Our analysis revealed several genomic regions with significant additive and non-additive genetic effects. Interestingly, these regions harbor genes, such as *SMAD7*, *SKAP1*, *ANKRD55*, *MRAP* and *MIS18A*, that are directly implicated in adipocyte differentiation, lipid metabolism, immune response, and insulin tolerance. We also performed a gene-set analysis to gain additional insights into the genetic architecture of visceral fat deposition. Our analysis revealed gene pathways and molecular mechanisms related to cell metabolism, cell signaling, and immune response, among others. We also assessed the genetic link between visceral fat and metabolic disorders. Notably, one region on BTA20, which harbors the gene *ANKRD55*, implicated in adipocyte differentiation and insulin resistance, showed significant effects on both visceral fat accumulation and displaced abomasum. Overall, our study suggests that visceral fat deposition in dairy cows is controlled by both additive and non-additive genetic factors. The genetic link between visceral fat accumulation and metabolic disorders deserves further investigation.

## Data Availability

The original contributions presented in the study are included in the article/Supplementary Material, further inquiries can be directed to the corresponding author.
